# Achieving surprise and play in unexpected places: clowns as artistic resources in oncology and palliative care

**DOI:** 10.3389/fpsyg.2026.1774266

**Published:** 2026-02-11

**Authors:** Susana Carnero-Sierra

**Affiliations:** 1 Basic Psychology Area, Psychology Department, Oviedo University, Oviedo, Spain; 2 Clowntigo: Healthcare Clowns, Asturias, Spain

**Keywords:** arts, clown doctors, healthcare clown, medical clown, oncology, palliative care

## 1 Introduction

A clown is an artistic figure who is always surrounded by the unexpected and who knows how to make the most of every annoyance. They are performers who provoke failures but also overcome them by playing with imagination and poetics. The clown is a character firmly situated in the present who always exhibits a contrast, either through their image or their actions, consequently achieving laughter and humor. Approximately 50 years ago, they began moving from the stage to visit places where healthcare was offered (Spitzer, 2006), thanks to their ability to listen sensitively to people and communicate creatively in both verbal and nonverbal ways.

Clowns are also symbols of human vulnerability who empathize with patient uncertainty and cope with and fight stress, anxiety, and pain (Fusetti et al., 2022). Thus, they evolved to become known as clown doctors, hospital clowns, or healthcare or therapeutic clowns. The effect of these professionals has been evaluated and studied in various populations and medical contexts, confirming their impact and supporting this type of intervention, and they are now established figures in the healthcare system (Dionigi et al., 2012).

Professional clown visitors reduce the anxiety and stress levels of hospitalized children and their families. Dionigi et al. (2014) observed decreased levels of preoperative anxiety in children, while Melvin et al. (2025) reported positive effects in psychiatric units for adolescents. Several reviews and meta-analyses have validated the general effect of modulating stress and anxiety (Sridharan and Sivaramakrishnan, 2016; Kasem et al., 2023; Lopes-Junior et al., 2020; Wang et al., 2024). Xin et al. (2024) also reported benefits in pain perception and cognitive behavioral issues, specifically in the context of children with autism. A recent study comparing a large sample of hospitalized pediatric patients with control groups found decreased levels of pain and crying time and improved levels of physical stress markers, such as increased oxytocin levels and lower cortisol levels (Sánchez et al., 2024).

Initially linked to pediatric healthcare, hospital clown organizations have expanded their scope to adult and senior care settings (Dionigi and Canestrari, 2016), and, with adaptations of their approach and routines, they are currently used in dementia care. Because of advanced age or illnesses associated with the aging process, clowns in senior healthcare increasingly connect with end-of-life contexts and are also beginning to be integrated into adult oncology and palliative care units.

## 2 Is there room for clowns in oncology and palliative care?

Healthcare clowns are affectionate and attentive artists who act in the present, channeling emotions and stimulating their audience with surprise, but without generating agitation or nervousness (Auerbach, 2017). Professional hospital clowns can identify when the moment to perform their interventions arises (Pinna et al., 2018), prioritizing respect and attentive listening in all circumstances while remaining extremely sensitive to patient silence, anger, or frustration.

Their aim is to support each individual, making each person feel valued and special, enriching their interactions with resources ranging from music and magic to juggling and poetry, which allows their audience to process grief and difficulties related to illness, including severe conditions in oncology and palliative care.

As mentioned, research experiences have supported the appropriateness of the presence of specialized clowns in these sensitive spaces. In pediatric oncology, findings include lower levels of the stress biomarker salivary cortisol, lower fatigue scale scores (Lopes-Junior et al., 2020), and less pain and distress, along with enhanced calm during chemotherapy sessions (Arriaga et al., 2020; Kurudirek and Arikan, 2020). Santarpia et al. (2023) addressed the spiritual domains that clowns can manage during their visits to pediatric palliative care units. Also in pediatric palliative care, healthcare clowns play a significant role in modulating perceptions of hair loss or in enabling children to choose the clown to express emotions that the child does not usually show their parents, such as sadness (Valdebenito, 2021, 2022; Valdebenito and Sánchez, 2021).

Studies have shown that hospital clowns also work well with the adult population. Dionigi and Canestrari (2016) confirmed that clown interventions enhance well-being by reducing the intensity of emotions such as stress and anxiety. Casellas-Grau et al. (2020) studied a sample of adult oncology patients and their relatives, finding improved levels of fatigue, discouragement, anxiety, boredom, sadness, anger, laughter, happiness, worry, disappointment, and fear.

## 3 Functions of healthcare clowns in oncology and palliative care settings

Understanding the specific way in which clowns achieve these outcomes requires consideration of the precise functions that benefit the psychosocial well-being of patients, their families, and staff. The following five functions are not limited to oncology and palliative care; however, it is in these services that they can play a particularly outstanding role.

### 3.1 Surprise

Clowns operate primarily by reacting from the logic of emotions, and the first emotion present in an encounter with a clown is surprise (Linge, 2011). The salience of a clown's presence serves as a powerful stimulus capable of distracting from fear, anxiety, stress, uncertainty, and even certain degrees of pain (Fusetti et al., 2022; Ding et al., 2022). Surprise and the subsequent emotions it generates, such as joy and tenderness, are incompatible with simultaneously experiencing negative emotional states.

Clowns in hospitals are highly unexpected interlocutors. Novelty, naturally linked to surprise, is a powerful property of stimuli that directly captures attention (Horstmann and Herwig, 2016). Surprising events generate intense memories, associated in this case with the moment a clown appears.

Linge (2011) described the surprise associated with the presence of a clown as a starting point for establishing a connection or a moment of insight. This initial surprise can act as a direct way to unlock other emotions and prepare patients for play and imagination (Linge, 2008).

### 3.2 Imagination and play

A fundamental aspect of a clown's basic training is broadening the capacity to enhance unexpected ways of responding to stimuli or solving problems. This imaginative attitude is in constant use and lived naturally. Santos et al. (2021) claimed that interaction with clowns encourages patients to establish new, clever, and creative reevaluations about life needs and adaptations (Anes and Obi, 2014).

This can be expressed by the term “playfulness”, which refers to the disposition to find new meanings in everyday or normative objects or situations, accompanied by feelings of joy, fun, and spontaneity (Bozionelos and Bozionelos, 1999; Dionigi et al., 2025). Playfulness is a measurable concept (Barnett, 1990; Lau et al., 2025) and is understood as a basic and essential seed of creativity that is inherent in the capacity to adapt and learn at any age.

For a clown, play is not infantilization but rather an imaginative strategy full of humanity, warmth, enthusiasm, closeness, and, of course, empathy. A clown's main mission in a hospital is to construct a space for imagination, transforming that space through play. Together with the patient, clowns physically embody and develop the patient's creative, living stories (Kontos et al., 2017), memories, and desires, moving them away from the hospital room to the seaside, mountains, forests, or anywhere else the patient wishes to go.

### 3.3 Humor to dissolve tension

Pinna et al. (2018) conducted a systematic review of the use of humor in palliative care, observing a list of useful functions of humor: transforming space, relieving moments of despair, distancing oneself from the medical context and situations of pain, handling and expressing personal vulnerability, releasing anxiety, stress, and fear, enhancing self-esteem, arousing imagination, and promoting positive remembrance. This description of humor aligns with the interventions of medical clowns.

Clowns can find laughter on many occasions, breaking the tension and stress present in these spaces by using the unexpected contrast of humor, but never forcing it. What happens is always done with sensitivity and their ability to build a shared expression.

### 3.4 Communication and expression

Healthcare clowns employ several skills to enhance communication, which is especially beneficial when interacting with patients who have verbal impairments. This is illustrated first of all by their proficiency in using non-verbal expressiveness and gestures. Second, communication happens using music, either by bringing songs that are meaningful to patients in the hospital or by leveraging their high sensitivity to listen to and respond to the rhythms of the space, adjusting to the tempo of the interaction established between interlocutors. A third benefit lies in establishing communication through the gaze and basic emotional expression, which also stimulates the expression of others' emotions.

This becomes especially relevant when considering older adults receiving oncology and palliative care who may also be experiencing dementia or speech problems derived from neurological damage. This was demonstrated using an ethogram of micro-gestures, which found a significant increase in communicative intent among the older population in interactions with healthcare clowns (Pérez-González and Carnero-Sierra, 2023). Clowns have excellent skills for capturing and interpreting subtle signals from people and their environments, allowing them to adapt their proposals and share in the experience of others. They also have outstanding potential to understand and respond to the emotional states of others, acting by focusing on the present and integrating elements provided by their artistic tools to enhance participant engagement (Roberts et al., 2024).

### 3.5 Support

The hospital clown is clearly a supportive figure (Santos et al., 2021), providing emotional release and psychological relief (Valdebenito and Sánchez, 2021), as shown by the various measures of anxiety and stress previously mentioned. A clown's intervention serves as an acknowledgment of the individual, distinguishing them from their illness and giving priority to the patient's initiative and right to make their own decisions (Warren and Spitzer, 2011). In the specific context of the end-of-life process, clowns work as a channel to celebrate both life and memories.

In addition, clown support also extends to staff. Brief encounters with members of staff provide relief through distraction and playfulness (Gomberg et al., 2020), particularly for mental health professionals, who are at the end of the care chain.

Additionally, clowns are a source of artistic tools that enrich and support healthcare teams, offering great opportunities for physical and cognitive stimulation, and encouraging initiative when performing sensorial, speech, or movement rehabilitation exercises. Furthermore, clowns encourage eating, taking medication, or accepting invasive practices such as injections (Javed et al., 2021). Storytelling, the transformative and elaborate function of play, physical theater, and the grammar of movement, music mixed with presence, juggling, magic tricks, objects, and theater, are tools at the service of clowns in their interventions. They can collaborate with psychosocial professionals in oncological and palliative settings.

## 4 Discussion

Healthcare clowns surprise and stir emotions in settings of grief and pain through humor, but are also attuned to opportunities to channel and process negative emotions through play and imagination. They are effective figures of support and reminders of the right to imagine and play, regardless of condition or age. This makes the clown duo a highly versatile tool for addressing needs that arise in the contexts of oncology or palliative care, whether with pediatric or adult patients, celebrating all possibilities and reinforcing every achievement and attempt at communication.

Another important point is the capacity of clown interventions to generate a strong psychological impact in a relatively short amount of time. A single performance can create intense memories due to its significance and unexpected presence. Another essential aspect of clowns is their ability to work in the absence of verbal language, always respecting reality and individuality while using their creativity to encourage patients' expression.

The clown is an interlocutor who naturally experiences all facets of being human. Ultimately, this is the essence of the clown: their poetic presence and actions transform the place and channel the emotional circumstances of extreme moments (Santarpia et al., 2023). Poetics also brings psychological comfort. Being a clown for patients experiencing grave circumstances is more than just dressing up and wearing a red rubber nose: a clown is a human being who sublimates pain through art and humanity.

## References

[B1] Anes L. Obi M. (2014). Hospital clowning as play stimulus in healthcare. Children 1, 374–389. doi: 10.3390/children1030374 27417485 PMC4928740

[B2] Arriaga P. Melo A. S. Caires S. (2020). The effects of hospital clowning on physical and emotional states of pediatric patients during chemotherapy treatment. Child Youth Care Forum 49, 365–381. doi: 10.1007/s10566-019-09532-6

[B3] Auerbach S. (2017). Are clowns good for everyone? The influence of trait cheerfulness on emotional reactions to a hospital clown intervention. Front. Psychol. 8:1973. doi: 10.3389/fpsyg.2017.01973 29180976 PMC5693907

[B4] Barnett L. A. (1990). Playfulness: definition, design, and measurement. Play Cult. 3, 319–333.

[B5] Bozionelos N. Bozionelos G. (1999). Playfulness: Its relationship with instrumental and expressive traits. Pers. Individ. Dif. 26, 749–760. doi: 10.1016/S0191-8869(98)00207-4

[B6] Casellas-Grau A. Ochoa C. Lleras De Frutos M. Flix-Valle A. Rosales A. Gil F. (2020). Perceived changes in psychological and physical symptoms after hospital clown performances in a cancer setting. Arts Health 13, 189–203. doi: 10.1080/17533015.2020.1744172 32223531

[B7] Ding Y. Yin H. Wang S. Meng Q. Yan M. Zhang Y. . (2022). Effectiveness of clown intervention for pain relief in children: A systematic review and meta-analysis. J. Clin. Nurs. 31, 3000–3010. doi: 10.1111/jocn.16195 34985166

[B8] Dionigi A. Canestrari C. (2016). Clowning in health care settings: The point of view of adults. Eur. J. Psychol. 12, 473–488. doi: 10.5964/ejop.v12i3.1107 27547261 PMC4991052

[B9] Dionigi A. Canestrari C. Fermani A. (2025). Caring with humor: How cognitive flexibility enhances humor and playfulness in clown doctors. J. Humanist. Psychol. (in press). https://journals.sagepub.com/toc/JHP/0/0?startPage=6&pageSize=10 (Accessed February 2, 2026).

[B10] Dionigi A. Flangini R. Gremigni P. (2012). “Clowns in hospitals,” in Humor and Health Promotion, ed. P. Gremigni (Hauppauge, NY: Nova Science Publishers), 213–228.

[B11] Dionigi A. Sangiorgi D. Flangini R. (2014). Clown intervention to reduce preoperative anxiety in children and parents: a randomized controlled trial. J. Health Psychol. 19, 369–380. doi: 10.1177/1359105312471567 23362335

[B12] Fusetti V. Re L. Pigni A. Tallarita A. Cilluffo S. Caraceni A. T. . (2022). Clown therapy for procedural pain in children: a systematic review and meta-analysis. Eur. J. Pediatr. 181, 2215–2225. doi: 10.1007/s00431-022-04440-9 35294645

[B13] Gomberg J. Raviv A. Fenig E. Meiri N. (2020). Saving costs for hospitals through medical clowning: A study of hospital staff perspectives on the impact of the medical clown. Clin. Med. Insights Pediatr. 14:1179556520909376. doi: 10.1177/1179556520909376 32214864 PMC7065276

[B14] Horstmann G. Herwig A. (2016). Novelty biases attention and gaze in a surprise trial. Atten. Percept. Psychophys. 78, 69–77. doi: 10.3758/s13414-015-0995-1 26486643

[B15] Javed T. Khan A. S. Jarral N. A. Taqi Z. Raza M. Shahid Z. (2021). Medical clowning: A cost-effective way to reduce stress among children undergoing invasive procedures. Cureus 13:e18886. doi: 10.7759/cureus.18886 34804732 PMC8599118

[B16] Kasem A. Meiri N. Pillar G. (2023). Medical clowning in hospitalized children: a meta-analysis. World J. Pediatr. 19, 1055–1061. doi: 10.1007/s12519-023-00720-y 37058203 PMC10533584

[B17] Kontos P. Miller K. L. Mitchell G. J. Stirling-Twist J. (2017). Presence redefined: The reciprocal nature of engagement between elder-clowns and persons with dementia. Dementia 16, 46–66. doi: 10.1177/1471301215580895 25908500 PMC4956469

[B18] Kurudirek F. Arikan D. (2020). Effects of therapeutic clowning on pain and anxiety during intrathecal chemotherapy in Turkey. J. Pediatr. Nurs. 53, e6–e13. doi: 10.1016/j.pedn.2020.01.015 32057641

[B19] Lau C. Brauer K. Quilty L. Chiesi F. Saklofske D. Proyer R. T. (2025). Revisiting the English Short Measure for Adult Playfulness (SMAP): An investigation of reliability, validity, and cross-cultural comparisons. J. Pers. Assess. 107, 256–264. doi: 10.1080/00223891.2024.2390004 39255359

[B20] Linge L. (2008). Hospital clowns working in pairs-in synchronized communication with ailing children. Int. J. Qual. Stud. Health Well-being 3, 27–38. doi: 10.1080/17482620701794147

[B21] Linge L. (2011). Joy without demands: Hospital clowns in the world of ailing children. Int. J. Qual. Stud. Health Well-being 6:5899. doi: 10.3402/qhw.v6i1.5899 21394247 PMC3053039

[B22] Lopes-Junior L. C. Silveira D. S. Olson K. Bomfim E. O. Veronez L. C. Santos J. C. . (2020). Clown intervention on psychological stress and fatigue in pediatric patients with cancer undergoing chemotherapy. Cancer Nurs. 43, 290–299. doi: 10.1097/NCC.0000000000000690 30801267

[B23] Melvin G. Hofmann J. L. Pavlou C. Lu S. Verstandig S. Taylor A. . (2025). The impacts of a clown doctor program on an adolescent psychiatric unit: A mixed methods investigation. Child Psychiatry Hum. Dev. 56, 142–152. doi: 10.1007/s10578-023-01545-6 37227620 PMC11828834

[B24] Pérez-González J. Carnero-Sierra S. (2023). Observación e impacto específico de las intervenciones de payasos de hospital en establecimientos residenciales para la tercera edad. Más Calidad 28, 35–60. Spanish.

[B25] Pinna M. A. C. Mahtani-Chugani V. Sánchez Correas M. A. Sanz Rubiales A. (2018). The use of humor in palliative care: a systematic literature review. Am. J. Hosp. Palliat. Care 35, 1342–1354. doi: 10.1177/1049909118764414 29587520

[B26] Roberts S. J. Luckett T. Ivynian S. DiGiacomo M. (2025). Elder clowning interventions for persons with dementia in long-term care: a systematic review and metasynthesis of qualitative research. Dementia 2, 192–214. doi: 10.1177/14713012251366738 41387393 PMC12701092

[B27] Sánchez J. C. Porras G. L. Torres M. A. Olaya J. C. García A. M. Muñoz L. V. . (2024). Effects of clowning on anxiety, stress, pain, and hormonal markers in paediatric patients. BMC Pediatr. 24:728. doi: 10.1186/s12887-024-05211-1 39533218 PMC11558874

[B28] Santarpia A. Romani-Cesaro M. Simonds C. (2023). Hospital clown narratives in pediatric palliative care. J. Humanist. Psychol. 63, 586–606. doi: 10.1177/0022167819850002

[B29] Santos F. R. D. Pinto S. Pessalacia J. D. R. Luchesi B. M. Silva L. A. D. Marinho M. R. (2021). Effects of clown activities on patients eligible for palliative care in primary health care. Rev. Bras. Enferm. 74:e20200431. doi: 10.1590/0034-7167-2020-0431 34346954

[B30] Spitzer P. (2006). Hospital clowns—modern-day court jesters at work. Lancet 368, S34–S35. doi: 10.1016/S0140-6736(06)69919-4

[B31] Sridharan K. Sivaramakrishnan G. (2016). Therapeutic clowns in pediatrics: a systematic review and meta-analysis of randomized controlled trials. Eur. J. Pediatr. 175, 1353–1360. doi: 10.1007/s00431-016-2764-0 27605131

[B32] Valdebenito V. (2021). The passage from one place to other: hospital clowns in a pediatric palliative care ward in Chile. Acta Bioeth. 27, 201–210. doi: 10.4067/S1726-569X2021000200201

[B33] Valdebenito V. (2022). Integration of healthcare clowns into pediatric palliative care: A bridge between life and death. Int. J. Integr. Care 22:10. doi: 10.5334/ijic.6506 36447461 PMC9673614

[B34] Valdebenito V. Sánchez R. (2021). Effects of hospital clowns on the quality of life of people in a Paediatric Oncology Palliative Care Unit. Med. Paliativa 28, 230–235.

[B35] Wang L. Zhu J. Chen T. (2024). Clown care in the clinical nursing of children: a meta-analysis and systematic review. Front. Pediatr. 12:1324283. doi: 10.3389/fped.2024.1324283 38590768 PMC10999578

[B36] Warren B. Spitzer P. (2011). Laughing to longevity - the work of elder clowns. Lancet 378, 562–563. doi: 10.1016/S0140-6736(11)61280-4 21847805

[B37] Xin G. Yingping F. Yue C. Jiaming W. Xue H. (2024). Application of clown care in hospitalized children: A scoping review. PLoS ONE 19:e0313841. doi: 10.1371/journal.pone.0313841 39700221 PMC11658477

